# Plant Essential Oils Inhibit Growth and Histamine Production of *Aeromonas hydrophila* Isolated from Skipjack Tuna

**DOI:** 10.3390/foods15132256

**Published:** 2026-06-23

**Authors:** Yifan Ren, Ruixue Cao, Zhunyao Zhu, Xiaopeng Zou, Longqi Gu, Xiangzhong Zhao

**Affiliations:** 1 School of Food Sciences and Engineering, Qilu University of Technology, Shandong Academy of Sciences, Jinan 250353, China; 15288886736@163.com (Y.R.); 13655317129@163.com (R.C.); 18366260356@163.com (Z.Z.); 18297817593@163.com (X.Z.); glq053088@163.com (L.G.); 2 Shandong Modern Marine Industry Technology Innovation Center, Tuna Processing, Qingdao 266400, China

**Keywords:** skipjack tuna, histamine-producing bacteria, *Aeromonas hydrophila*, plant essential oils, histamine inhibition

## Abstract

The accumulation of histamine in fish products represents a significant food safety issue, particularly in skipjack tuna (*Katsuwonus pelamis*), due to its elevated histidine content. This study sought to isolate histamine-producing bacteria from skipjack tuna and assess the inhibitory effects of six plant-derived essential oils on bacterial proliferation and histamine synthesis. Seven bacterial isolates were obtained and screened, with histamine concentrations quantified via high-performance liquid chromatography (HPLC) following dansyl chloride derivatization. The isolate exhibiting the highest histamine production (1.2 ± 0.2 mM) was identified as *Aeromonas hydrophila* through 16S rDNA sequencing. Essential oils were administered to bacterial cultures prior to histamine quantification, and their minimum inhibitory concentrations (MICs) and minimum bactericidal concentrations (MBCs) were determined in vitro. Among the tested oils, oregano and cinnamon demonstrated the strongest antibacterial activity, with MIC and MBC values below 1 mg/mL. Scanning electron microscopy analysis revealed pronounced structural damage to bacterial cells treated with these oils. At the MBC, histamine production was entirely suppressed; at half the MBC, histamine synthesis was reduced by more than 90%, whereas lower concentrations yielded moderate inhibition ranging from 15% to 22%. These findings suggest that selected essential oils, notably oregano and cinnamon, possess considerable potential as natural preservatives to reduce histamine formation in skipjack tuna. However, further investigation is necessary to confirm their effectiveness under practical storage conditions.

## 1. Introduction

Skipjack tuna (*Katsuwonus pelamis*) represents a significant pelagic species within commercial fisheries. Owing to its elevated protein content and distinctive flavor profile [1,2,3], it is extensively utilized in the manufacture of canned, dried, raw consumption, and prepared seafood products [4,5]. Notably, skipjack tuna contains a high concentration of histidine [6]. Inadequate temperature regulation during capture, transportation, storage, and processing can facilitate the accumulation of histamine, thereby heightening the risk of histamine poisoning [7]. Histamine poisoning typically arises from the microbial decarboxylation of free histidine present in fish muscle, leading to histamine formation [8]. This risk is compounded by the fact that histamine, once formed, is not readily eliminated through heat treatment [9]. Consequently, mitigating histamine formation during the processing and storage of skipjack tuna is imperative to ensure the safety of the fish and its derived products.

The formation of histamine is primarily driven by histidine decarboxylase enzymes produced by histamine-producing microorganisms [10]. During capture and processing, skipjack tuna may be contaminated by microorganisms originating from the environment, equipment, fish surfaces, and endogenous sources. Under favorable storage conditions, histamine-producing bacteria can proliferate rapidly, converting free histidine into histamine [11]. Prior research has identified *Morganella* spp., certain Enterobacteriaceae members, and *Aeromonas* spp. as contributors to histamine and the formation of other biogenic amines in aquatic products [12,13]. Given that the predominant amine-producing bacteria vary according to fish origin, processing environments, and storage conditions, it is challenging to precisely identify the principal risk bacteria in specific samples based solely on existing literature. Therefore, isolating, screening, and characterizing strains with potent histamine-producing capabilities from skipjack tuna samples is essential for subsequent targeted inhibition studies [14].

Currently, histamine control in aquatic products predominantly relies on low-temperature storage, cold-chain logistics, and stringent hygienic practices, which are critical for retarding microbial proliferation. However, maintaining uninterrupted low-temperature conditions is often difficult during long-distance fishing, distribution, and conventional processing. Should histamine-producing bacteria multiply extensively during the initial storage phase, the risk associated with preformed histamine remains difficult to fully mitigate, even if low-temperature conditions are later reinstated [15]. Hence, beyond temperature control, the development of supplementary strategies to inhibit the growth of histamine-producing bacteria and their histamine synthesis is of practical importance. Recently, natural antimicrobial agents have attracted considerable interest in food preservation due to their broad availability, high consumer acceptance, and promising applications in enhancing food safety [16].

Plant essential oils constitute volatile mixtures of secondary metabolites, typically comprising phenols, aldehydes, alcohols, and terpenes [17]. Essential oils extracted from oregano, cinnamon, clove, and rosemary have exhibited antimicrobial properties against a range of food spoilage and pathogenic bacteria. Their antimicrobial mechanisms are proposed to include disruption of cell membrane integrity, modification of membrane permeability, interference with enzymatic activities, and impairment of cellular metabolic processes [18,19]. Despite extensive research on plant essential oils as natural antimicrobial agents in food preservation [20,21], their effects on histamine-producing bacteria isolated directly from skipjack tuna remain inadequately characterized [10,22]. Notably, comparative data on the antibacterial efficacy of various essential oils against high histamine-producing bacterial isolates and their impact on histamine accumulation are scarce [10,23]. Consequently, the present study aims to address this gap by isolating histamine-producing bacteria from skipjack tuna, selecting the strain with the highest histamine production for targeted investigation, and systematically evaluating the inhibitory effects of six plant essential oils on bacterial growth, cellular morphology, and final histamine accumulation. The outcomes of this research are anticipated to contribute a scientific foundation for the development of natural interventions to mitigate histamine formation during the storage and processing of skipjack tuna products.

## 2. Materials and Methods

### 2.1. Skipjack Tuna Samples

Skipjack tuna specimens were procured from Qingdao Hengxin Aquatic Products Co., Ltd. (Qingdao, China) and transported to the laboratory under refrigerated conditions maintained at 4 °C. A total quantity of 10 kg of frozen whole skipjack tuna was obtained. The transportation duration was approximately 20 h. Upon arrival, samples were aseptically processed for microbial isolation.

### 2.2. Reagents

Trichloroacetic acid (TCA), 1,7-diaminoheptane (≥98%), and dansyl chloride (≥99%), utilized for the quantification of histamine in skipjack tuna, were sourced from Aladdin Reagent Co., Ltd. (Shanghai, China). Acetonitrile (HPLC grade, ≥99.9%), acetone, sodium chloride, and D-(+)-glucose were procured from Hushi Reagent Co., Ltd. (Shanghai, China). Histamine dihydrochloride and 2.5% glutaraldehyde were obtained from Yuanye Bio-Technology Co., Ltd. (Shanghai, China).

Peptone, yeast extract, L-histidine, agar, sodium chloride, calcium carbonate, MRS broth, nutrient agar medium, and bromocresol purple, employed for bacterial isolation from skipjack tuna and subsequent cultivation, were purchased from Beijing Aoboxing Bio-Technology Co., Ltd. (Beijing, China). Purified water used throughout the experiments was Wahaha drinking purified water (Hangzhou Wahaha Group Co., Ltd., Hangzhou, China).

Clove essential oil (≥99%), rosemary essential oil (≥99%), oregano essential oil (≥99%), patchouli essential oil (≥99%), perilla essential oil (≥99%), and cinnamon essential oil (≥99%), applied in antibacterial susceptibility assays, were acquired from Jiangxi Hairui Natural Plant Co., Ltd. (Ji’an, China). Sterile antimicrobial susceptibility disks, measuring 6 mm in diameter, were obtained from Bikeman Biotechnology Co., Ltd. (Changsha, China).

### 2.3. Isolation of Histamine-Producing Bacteria from Skipjack Tuna

Histamine-producing bacteria were isolated following the protocol described by Bover et al. [24], with minor modifications. In brief, dorsal muscle samples of refrigerated skipjack tuna, with the skin intact, were weighed and combined with sterile physiological saline. The mixture was homogenized thoroughly using sterile homogenization bags and a homogenizer.

The homogenate was serially diluted using 0.1 mol/L phosphate-buffered solution to achieve dilutions of 10^−2^, 10^−3^, and 10^−4^. Aliquots of these dilutions were plated onto plate count agar (PCA) and incubated at 30 °C for 36 h. Colonies exhibiting distinct morphological characteristics were selected and purified through two to three successive subcultures. The purified isolates were subsequently inoculated onto Niven’s biogenic amine screening medium. Single colonies that induced a color change in the medium surface to blue or bluish-purple were selected and further purified through two to three additional subcultures. Following incubation at 30 °C for 36 h, the strains were stored at 4 °C until further analysis.

### 2.4. Identification of Histamine-Producing Bacteria in Skipjack Tuna

Genomic DNA was extracted employing the alkaline lysis technique. The bacterial lysis solution was prepared by combining 1 μL of 0.2 M sodium hydroxide (NaOH) with 2.5 μL of 1% sodium dodecyl sulfate (SDS). In brief, 5 μL of this lysis solution was added to a 1.5 mL microcentrifuge tube containing an appropriate quantity of bacterial cell pellet. The mixture was gently agitated to minimize foaming and incubated at ambient temperature for 5 min. The lysis reaction was subsequently terminated by the addition of 200 μL of double-distilled water (ddH_2_O), and the resulting solution was utilized as the DNA template for polymerase chain reaction (PCR) amplification.

The bacterial 16S ribosomal RNA (rRNA) gene was amplified using universal primers 27F and 1492R, with sequences 5′-AGAGTTTGATCCTGGCTCAG-3′ and 5′-GGTTACCTTGTTACGACTT-3′, respectively. PCR amplification was conducted in a total volume of 25 μL, comprising 10 μL of ddH_2_O, 0.5 μL each of primers 27F and 1492R, 12.5 μL of Taq PCR Master Mix, and 1.5 μL of the DNA template. The reaction mixture was gently mixed prior to amplification.

Amplification was performed using a Biometra 844-070-882 thermal cycler (Biometra GmbH, Göttingen, Germany) under the following conditions: initial denaturation at 94 °C for 3 min; 30 cycles consisting of denaturation at 94 °C for 45 s, annealing at 58 °C for 45 s, and extension at 72 °C for 2 min; followed by a final extension at 72 °C for 10 min. The amplified products were subsequently maintained at 4 °C.

PCR products were analyzed via agarose gel electrophoresis at 120 V and 120 mA for 20 min. Post-electrophoresis, the amplified DNA bands were visualized and documented using a Tanon 2500 gel imaging system (Tanon Science & Technology Co., Ltd., Shanghai, China).

PCR products exhibiting distinct target bands were subjected to Sanger sequencing. Initial sequencing was performed in a single direction. Sequences demonstrating clear chromatograms without overlapping peaks and of sufficient quality were utilized for further analysis; bidirectional sequencing was conducted when necessary. The obtained sequences were assembled and edited using DNA MAN software (Version 10, released 2022; Lynnon Biosoft (San Ramon, CA, USA), https://www.lynnon.com/dnaman.html, accessed 15 February 2026). The finalized 16S rRNA gene sequences were submitted to the National Center for Biotechnology Information (NCBI) database and compared against reference sequences using the Basic Local Alignment Search Tool (BLAST) online web server (https://blast.ncbi.nlm.nih.gov, accessed 15 February 2026). to ascertain the taxonomic identities of the bacterial isolates.

### 2.5. Quantification of Histamine

Histamine quantification was conducted following the protocols described by Sánchez-Parra et al. [25] and Zhong et al. [26], with minor modifications. The derivatization procedure for both histamine standard solutions and sample extracts was carried out as follows. In brief, 300 μL of each standard working solution was placed into a 2 mL centrifuge tube, to which 40 μL of 0.1 mol/L sodium hydroxide (NaOH) solution was added. Subsequently, 600 μL of dansyl chloride solution in acetone (10 mg/mL) was introduced. The mixture was vortexed thoroughly and incubated at 40 °C for 20 min in the dark to facilitate derivatization. Upon completion, 100 μL of ammonia solution was added to terminate the reaction. The mixture was vortexed again and maintained in the dark for an additional 30 min. Finally, 460 μL of acetonitrile was added, followed by vortexing; the solution was then filtered through a 0.22 μm organic-phase membrane filter and transferred into an injection vial for high-performance liquid chromatography (HPLC) analysis.

For sample preparation, the edible portions of aquatic product samples were homogenized thoroughly. A 5.0 g aliquot, measured with an accuracy of 0.01 g, was weighed into a 50 mL plastic centrifuge tube. To this, 312.5 μL of 1,7-diaminoheptane internal standard stock solution (10 mg/mL) and 12 mL of 5% (*w*/*v*) trichloroacetic acid aqueous solution were added. The mixture was vortexed for 2 min and centrifuged at 4000 rpm for 2 min. The supernatant was transferred into a 25 mL amber volumetric flask. The residue was subjected to a second extraction with 10 mL of 5% trichloroacetic acid aqueous solution under identical conditions. The combined supernatants were diluted to volume (25 mL) with 5% trichloroacetic acid solution and mixed thoroughly. An aliquot of the resulting extract was filtered through a 0.22 μm membrane filter prior to further analysis.

For the derivatization of the sample extract, 300 μL of the filtered extract was transferred into a 2 mL centrifuge tube. Given the presence of trichloroacetic acid in the extract, 40 μL of a 2 mol/L sodium hydroxide solution was added to adjust the pH to approximately 9.5. Subsequently, 600 μL of a dansyl chloride solution in acetone (10 mg/mL) was introduced. The mixture was thoroughly vortexed and incubated at 40 °C for 20 min in the absence of light. Upon completion of the reaction, 100 μL of ammonia solution was added to terminate the derivatization process. The mixture was vortexed again and kept in the dark for an additional 30 min. Finally, 460 μL of acetonitrile was added, the mixture was vortexed, filtered through a 0.22 μm organic-phase membrane filter, and transferred into an injection vial for high-performance liquid chromatography (HPLC) analysis. Histamine quantification was conducted using an HPLC system equipped with a UV detector (Shimadzu LC-20A, Shimadzu, Kyoto, Japan). Chromatographic separation was achieved on an Agilent Eclipse Plus C18 reversed-phase column (250 mm × 4.6 mm, 5 μm; Agilent Technologies, Santa Clara, CA, USA). The mobile phase comprised acetonitrile and water, with gradient elution performed according to the program detailed in Table 1. The flow rate was set at 0.8 mL/min, detection was carried out at a wavelength of 254 nm, the column temperature was maintained at 35 °C, and the injection volume was 20 μL. Histamine concentrations were determined using the internal standard method, wherein the calibration curve was constructed by plotting the peak area ratio of histamine to 1,7-diaminoheptane against known histamine concentrations.

Histamine concentrations in bacterial cultures were determined through high-performance liquid chromatography (HPLC) subsequent to derivatization with dansyl chloride. To achieve precise quantification, 1,7-diaminoheptane was employed as an internal standard. Bacterial isolates were cultivated in nutrient broth containing 1% (*w*/*v*) histidine at 30 °C for a duration of five days before undergoing extraction and derivatization procedures.

### 2.6. Preparation of Essential Oil Emulsions

Tween 80 was used as a nonionic surfactant. An aqueous solution containing 1% (*v*/*v*) Tween 80 was prepared, and the essential oils were mixed with the aqueous phase at a specified ratio. Essential oil emulsions were prepared by high-speed shearing and ultrasonic treatment and were freshly prepared before use. For brevity, oregano essential oil, cinnamon essential oil, clove essential oil, perilla essential oil, rosemary essential oil, and patchouli essential oil are abbreviated as OEO, CEO, CLEO, PEO, REO, and PaEO, respectively, in the figures.

### 2.7. Antibacterial Effects of Plant Essential Oils Against Histamine-Producing Bacteria

#### 2.7.1. GC–MS Analysis of Antibacterial Components in Essential Oils

The antibacterial components of the essential oils were analyzed by GC–MS according to the method of Prakhyath et al. [18], with slight modifications. Each natural plant essential oil was prepared as a 1 mg/mL solution using analytical-grade cyclohexane (Macklin, Shanghai, China) as the solvent. The solution was filtered through a polyethersulfone syringe filter with a pore size of 0.25 μm and transferred into chromatographic vials for analysis.

GC–MS analysis was performed using a gas chromatography–mass spectrometry system (TSQ 8000 Evo, Thermo Fisher Scientific, Guangzhou, China). Helium was used as the carrier gas at a flow rate of 1.5 mL/min. The injector temperature was set at 240 °C. A sample volume of 1.0 μL was automatically injected in splitless mode. The oven temperature was initially set at 50 °C and held for 2 min, then increased to 220 °C at a rate of 5 °C/min and held for 10 min. The temperature was further increased to 250 °C and held for 1 min. Compounds were identified by calculating the retention index (RI). The RI values were calculated using GC–MS data of n-alkanes (C9–C30) and compared with values reported in the literature. An RI deviation within ±30 was considered acceptable to improve the reliability of compound identification.

#### 2.7.2. Antibacterial Screening

Antibacterial screening was performed using the disk diffusion method [27] and related literature methods, with appropriate modifications for the preliminary evaluation of the antibacterial activity of plant essential oils. Under the action of antibacterial substances, the growth of microorganisms on the surface of solid medium is inhibited, forming a clear circular zone. The diameter of this zone, expressed in millimeters, was used to evaluate the in vitro antibacterial activity of the tested substance against the target microorganism.

For preparation of the bacterial suspension, a single colony of each strain was inoculated into 10 mL of nutrient broth and cultured at 30 °C for 6 h. The bacterial suspension was adjusted to approximately 10^6^ CFU/mL, and 100 μL was inoculated onto plate count agar. Meanwhile, 20 μL of antibacterial solution was added onto each sterile 6 mm paper disk. Three filter paper disks were placed on each plate, followed by incubation at 30 °C for 24 h. After incubation, the diameters of the inhibition zones were measured. Essential oils with good antibacterial effects were selected for subsequent experiments. All experiments were performed in triplicate.

#### 2.7.3. Minimum Inhibitory Concentration and Minimum Bactericidal Concentration

The essential oils were diluted using the microdilution method. Briefly, 1.0 g of essential oil was accurately weighed, dissolved in 1% Tween 80, and diluted to 50 mL. The mixture was shaken thoroughly and freshly prepared before use. This stock solution was serially diluted with 1% Tween 80 to obtain essential oil concentrations of 20.0, 10.0, 8.0, 6.0, 4.0, 2.0, and 1.0 mg/mL. The plant essential oils at different concentrations were added to sterile nutrient broth, resulting in final essential oil concentrations of 2.0, 1.0, 0.8, 0.6, 0.4, 0.2, and 0.1 mg/mL (Table 2).

A bacterial suspension cultured to the logarithmic phase was added to each tube at a volume of 50 μL. The bacterial suspension was adjusted to approximately 10^6^ CFU/mL, and the final bacterial concentration in the reaction system was approximately 10^5^ CFU/mL. Controls were also established, including a negative control containing 1% Tween 80 without bacterial suspension and a positive control containing 1% Tween 80 plus 50 μL of bacterial suspension. All treatments were performed in triplicate to ensure experimental reproducibility.

All test tubes were incubated at 30 °C for a duration of 24 h. The minimum concentration of essential oil that produced turbidity comparable to that of the negative control was designated as the minimum inhibitory concentration (MIC). Subsequently, culture broth from tubes at the MIC and higher concentrations was collected, and 100 μL aliquots were uniformly spread onto sterile nutrient agar plates. These plates were incubated at 30 °C for 24 h, after which bacterial colony formation was assessed. The lowest concentration at which no bacterial colonies were observed was defined as the minimum bactericidal concentration (MBC). MIC and MBC assessments were conducted in vitro employing the microdilution technique.

#### 2.7.4. Assessment of Essential Oil Effects on the Cellular Morphology of *Aeromonas hydrophila* via Scanning Electron Microscopy

The impact of essential oils on the cellular morphology of *Aeromonas hydrophila* (*A. hydrophila*) was examined using scanning electron microscopy (SEM), following the protocol described by Zhao et al. [28] with minor modifications. *A. hydrophila* cultures were grown to the logarithmic growth phase. Essential oils were then administered to experimental groups at concentrations corresponding to 0 × MIC, 1 × MIC, and 2 × MIC, respectively. The samples were incubated in a shaking incubator at 30 °C with agitation at 180 rpm for 3 h.

Post-treatment, bacterial cells were harvested by centrifugation at 8000 rpm for 10 min, and the supernatant was discarded. The cell pellets were fixed in 500 μL of glutaraldehyde at 4 °C for 12 h, followed by three washes with sterile phosphate-buffered saline (PBS). Dehydration of the bacterial samples was performed sequentially using graded ethanol solutions at concentrations of 30%, 50%, 70%, 80%, and 90%, each for 10 min, followed by two additional dehydration steps with 100% ethanol for 20 min each. Finally, samples were freeze-dried at −85 °C for 2 h and sputter-coated with gold. The morphological characteristics of *A. hydrophila* were then observed using SEM.

### 2.8. Data Analysis

All experimental measurements were conducted in triplicate, and results are presented as mean values ± standard deviation. Statistical analyses were performed using one-way analysis of variance (ANOVA) via IBM SPSS Statistics software version 26.0. A significance threshold was established at *p* < 0.05.

## 3. Results and Discussion

### 3.1. Isolation and Identification of Histamine-Producing Bacteria from Skipjack Tuna

Bacterial strains displaying distinct colony morphologies were isolated from the dorsal muscle tissue of skipjack tuna and subsequently subcultured to obtain pure cultures. A total of eleven bacterial isolates were obtained. These isolates were then inoculated onto Niven’s biogenic amine screening medium, where seven strains were preliminarily identified based on observable color changes in the medium.

To more precisely evaluate the histamine-producing potential of the isolates, they were cultured in nutrient broth supplemented with 1% histidine, and histamine concentrations were determined using high-performance liquid chromatography (HPLC). The histamine production levels of the seven isolates are summarized in Table 3. Among these, histamine was undetectable in four strains, whereas three strains produced measurable quantities. Two of these exhibited relatively low histamine production, while strain AH-2-TU demonstrated the highest histamine synthesis capacity.

**Figure 1:**
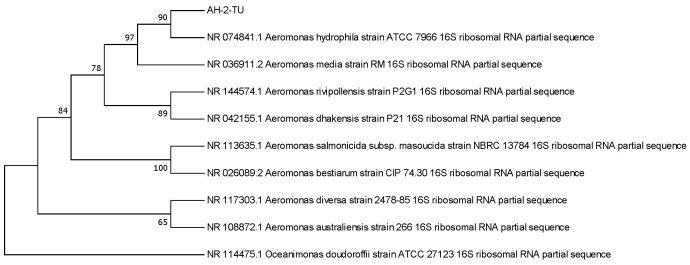
Phylogenetic tree of *A. hydrophila*.

Among the seven presumptive histamine-producing isolates, strain AH-2-TU produced the highest level of histamine and was therefore selected as the target strain for subsequent antibacterial and histamine-inhibition assays. To further determine its taxonomic identity, the 16S rRNA gene of strain AH-2-TU was amplified and sequenced. The obtained sequence was 1537 bp in length and has been deposited in GenBank under the accession number NR_074841.1. BLAST analysis against the NCBI database showed that the sequence shared the highest similarity with *Aeromonas hydrophila* strain ATCC 7966, with a query coverage of 99%, a sequence identity of 99.86%, and an E-value of 0.0 (Table 4). Phylogenetic analysis further showed that AH-2-TU clustered within the *Aeromonas hydrophila* clade, supporting its assignment to *A. hydrophila* based on 16S rRNA gene sequence analysis. The corresponding phylogenetic tree is presented in Figure 1.

However, it should be noted that species-level identification within the genus Aeromonas based solely on 16S rRNA gene sequencing has certain limitations, because closely related Aeromonas species may exhibit highly similar or nearly identical 16S rRNA gene sequences. Therefore, although the BLAST results and phylogenetic placement support the identification of AH-2-TU as *A. hydrophila*, this taxonomic assignment should be interpreted with caution. Additional approaches, such as multilocus sequence analysis, whole-genome sequencing, or biochemical identification, would provide higher resolution for confirming its species-level identity in future studies. In the present study, AH-2-TU was used as the representative high histamine-producing Aeromonas isolate for evaluating the inhibitory effects of plant essential oils.

### 3.2. Antibacterial Activity of Six Plant Essential Oils

Table 5 summarizes the results of the disk diffusion assay employed to assess the antibacterial efficacy of six plant essential oils against the target strain. Among the oils tested, oregano and cinnamon exhibited pronounced antibacterial activity, followed by clove oil. Perilla and rosemary oils demonstrated comparatively weaker inhibitory effects, whereas patchouli oil showed no significant antibacterial activity.

**Figure 2:**
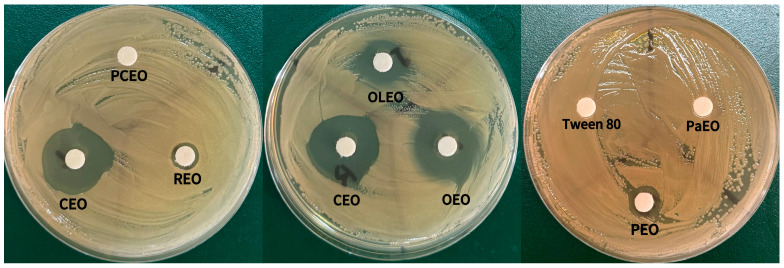
Visual observation of inhibition zones of six plant essential oils against *A. hydrophila*.

As shown in Figure 2, the disk diffusion assay revealed that essential oils derived from cinnamon, clove, perilla, oregano, and rosemary produced visible inhibition zones against *Aeromonas hydrophila* (*A. hydrophila*) strain AH-2-TU, indicating their antibacterial activity. Accordingly, these five essential oils were selected for further analysis using the microdilution assay. All tested plant essential oils exhibited growth inhibition at varying concentrations. The minimum inhibitory concentration (MIC) was defined as the lowest concentration at which no visible microbial turbidity was observed. Subsequently, for concentrations exhibiting no visible growth, plate counting was performed to ascertain the minimum bactericidal concentration (MBC), defined as the lowest concentration necessary to achieve complete microbial eradication. The MIC and MBC values for each essential oil are summarized in Table 6.

Among the essential oils evaluated, oregano and cinnamon demonstrated the highest efficacy, with both exhibiting MIC and MBC values below 1 mg/mL. The *A. hydrophila* strain AH-2-TU also showed considerable sensitivity to clove essential oil, which exerted a bactericidal effect at 1.0 mg/mL. Perilla and rosemary essential oils inhibited visible bacterial growth at concentrations ranging from 1 to 2 mg/mL; however, substantially higher concentrations were required to achieve bactericidal activity, with MBC values of 2 mg/mL and 4 mg/mL, respectively.

An antimicrobial agent is typically classified as bactericidal when the MBC/MIC ratio is less than or equal to 4, and bacteriostatic when this ratio exceeds 4 [29]. In the present investigation, cinnamon and oregano essential oils exhibited potent antibacterial activity, thereby contributing to the expanding theoretical framework regarding the antibacterial efficacy of essential oils as previously reported by Akdemir Evrendilek et al. [30]. Notably, these two essential oils maintained robust inhibitory effects across diverse biological conditions, underscoring their potential for development as natural broad-spectrum antibacterial agents.

### 3.3. Potential Antibacterial Mechanisms of Essential Oil Components

The histamine content in skipjack tuna is largely influenced by microbial proliferation during storage and transportation; thus, optimizing these conditions is critical to mitigating histamine accumulation. This study further assessed the impact of various plant essential oils on both the growth and histamine-producing capacity of the strain exhibiting the highest histamine production.

**Figure 3:**
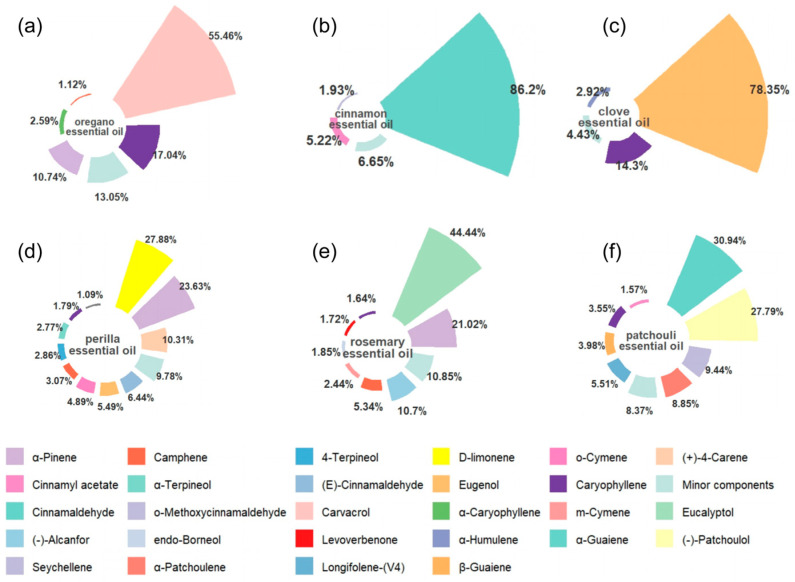
Relative percentages of the major volatile compounds identified in six plant essential oils by GC–MS analysis.

The GC–MS profiling, as presented in Figure 3, revealed notable variations in the predominant chemical components among the essential oils, which may underlie their differential antibacterial efficacy against *A. hydrophila* strain AH-2-TU.Oregano essential oil was chiefly characterized by a high concentration of carvacrol, accompanied by β-caryophyllene, α-pinene, and other minor constituents (panel a). Cinnamon essential oil was dominated by cinnamaldehyde (panel b), whereas clove essential oil primarily contained eugenol and caryophyllene (panel c). These essential oils exhibited either strong or moderate antibacterial activity, as evidenced by disk diffusion assays and minimum inhibitory concentration (MIC) and minimum bactericidal concentration (MBC) determinations. Notably, oregano and cinnamon essential oils demonstrated MIC and MBC values below 1 mg/mL, indicative of potent inhibitory effects against the target bacterial strain.

The pronounced antibacterial activity of oregano essential oil is likely attributable to its elevated carvacrol content. Carvacrol, a hydrophobic phenolic compound, is capable of interacting with the lipid bilayer of bacterial cell membranes, thereby altering membrane permeability, compromising membrane integrity, and inducing leakage of intracellular constituents, which collectively inhibit bacterial proliferation [31]. Similarly, cinnamaldehyde, the principal component of cinnamon essential oil, may exert antibacterial effects by disrupting cell membrane architecture, interfering with intracellular protein functions, and impairing metabolic enzyme activities [32]. These mechanisms may account for the relatively low MIC values observed for cinnamon essential oil in the current study. Clove essential oil, enriched in eugenol, is also postulated to exert antibacterial effects through disruption of membrane integrity and modulation of membrane-associated protein functions, which corresponds with its notable bactericidal activity against AH-2-TU.

Conversely, although perilla and rosemary essential oils contain volatile constituents such as terpenes and alcohols (panels d and e), their antibacterial efficacy was comparatively weaker than that of oregano, cinnamon, and clove essential oils. This observation suggests that the antibacterial potency of essential oils is not solely dependent on the concentration of major compounds but is also influenced by the nature of active constituents, potential synergistic or antagonistic interactions among components, and the inherent susceptibility of the target microorganism [33]. Patchouli essential oil, predominantly composed of patchouli alcohol, guaiene, and related compounds (panel f), did not produce a discernible inhibition zone under the experimental conditions, implying limited antibacterial activity against AH-2-TU or insufficient effective concentration and diffusion capacity in the assay.

Collectively, the compositional analysis via GC–MS and antibacterial evaluations indicate that the robust antibacterial properties of oregano and cinnamon essential oils may be ascribed to their enrichment in phenolic and aldehyde bioactive compounds, respectively. Variations in antibacterial efficacy among the tested essential oils likely result from the combined influence of the major active constituents, their relative proportions, diffusion characteristics, and the susceptibility profile of the target bacterium. To elucidate the precise mechanisms underlying the antibacterial effects of these essential oils against *A. hydrophila*, further investigations involving isolated active compounds, assessments of membrane permeability alterations, and analyses of intracellular content leakage are warranted.

### 3.4. Effects of Selected Essential Oils on Histamine Production

**Figure 4:**
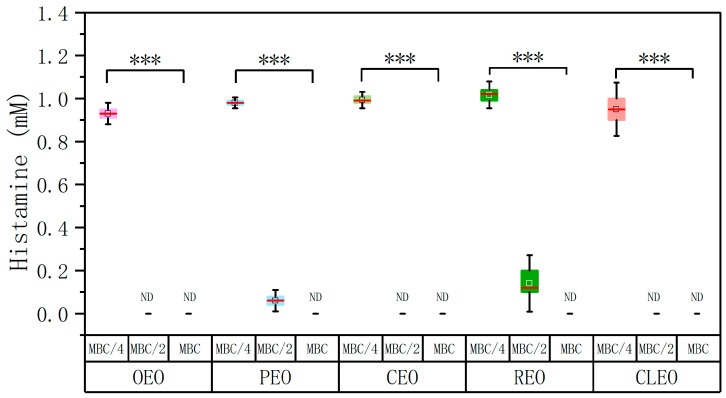
Effects of five plant essential oils at 1/4 MBC, 1/2 MBC, and MBCs on histamine production by *A. hydrophila*. Data are presented as box-and-whisker plots. All pairwise comparisons exhibited extremely significant differences (*p* < 0.001). Note: Distinct colors correspond to different essential oil treatments. The symbol *** denotes a highly significant difference at *p* < 0.001, while ND indicates the absence of detectable data.

The impact of five potent plant essential oils on the histamine-producing capacity of *A. hydrophila* AH-2-TU was systematically investigated. The bacterial strain was exposed to each essential oil at three distinct concentrations: the minimum bactericidal concentration (MBC), half the MBC (MBC/2), and one-quarter of the MBC (MBC/4). Following a 24 h incubation period, histamine levels within the culture medium were quantified. As illustrated in Figure 4, the inhibitory effects of the essential oils on histamine synthesis varied according to concentration, exhibiting a clear dose-dependent relationship.

At the MBC, all five essential oils effectively eradicated or suppressed the growth of *A. hydrophila* AH-2-TU, resulting in undetectable histamine levels in the culture system. This outcome suggests that histamine production is effectively halted when bacterial proliferation is controlled, corroborating the established notion that biogenic amine formation is largely contingent upon microbial growth and the activity of associated decarboxylase enzymes [34]. Conversely, at the lowest concentration tested (MBC/4), the inhibitory influence on histamine production was substantially diminished, with only a 15–22% reduction observed. This indicates that sublethal concentrations of essential oils are insufficient to significantly impair the histamine-producing capability of the strain.

Notably, at the intermediate concentration (MBC/2), all five essential oils demonstrated pronounced suppression of histamine synthesis. Specifically, oregano, cinnamon, and clove essential oils completely inhibited histamine production, whereas perilla and rosemary essential oils achieved reductions of 92% and 93%, respectively. This potent inhibitory effect may be attributed to the proximity of the MBC/2 concentrations to the minimum inhibitory concentrations (MICs) of the respective oils. For instance, the MBC/2 values for oregano, perilla, and rosemary essential oils approximated their MICs, while those for cinnamon and clove exceeded their MICs. Consequently, these concentrations were sufficient to restrict bacterial growth effectively, thereby further diminishing histamine formation. These findings suggest that the MBC/2 concentration represents a promising candidate for subsequent validation studies within skipjack tuna food matrices.

To control for potential confounding effects of the emulsifier Tween-80, a corresponding negative control was incorporated. The results indicated that Tween-80 did not significantly influence histamine detection, confirming that the observed inhibitory effects on histamine production were attributable primarily to the essential oils themselves rather than to emulsifier interference. The findings indicate that the chosen plant essential oils effectively suppressed bacterial proliferation and markedly decreased histamine synthesis. These results offer empirical support for their prospective use in mitigating histamine-associated hazards in aquatic food products.

It should be noted that this experiment was designed as an endpoint assessment of histamine accumulation rather than a kinetic analysis of histamine biosynthesis. The initial bacterial population in the reaction system was approximately 10^5^ CFU/mL. At the MBC, no recoverable colonies were observed according to the definition of MBC, which explains the undetectable histamine levels. However, final viable populations at sub-MBC treatments, residual histidine concentrations, and time-course histamine formation were not determined in the present study. Therefore, the observed decrease in histamine should not be interpreted as direct evidence that essential oils inhibited histidine decarboxylase activity. Instead, the results indicate that selected essential oils reduced endpoint histamine accumulation, mainly under conditions that inhibited or strongly restricted bacterial growth. Further studies using time-course sampling, residual histidine analysis, and enzyme activity assays are needed to distinguish between growth inhibition and direct inhibition of histidine decarboxylase.

### 3.5. Effects of Selected Essential Oils on the Cell Morphology of A. hydrophila

**Figure 5:**
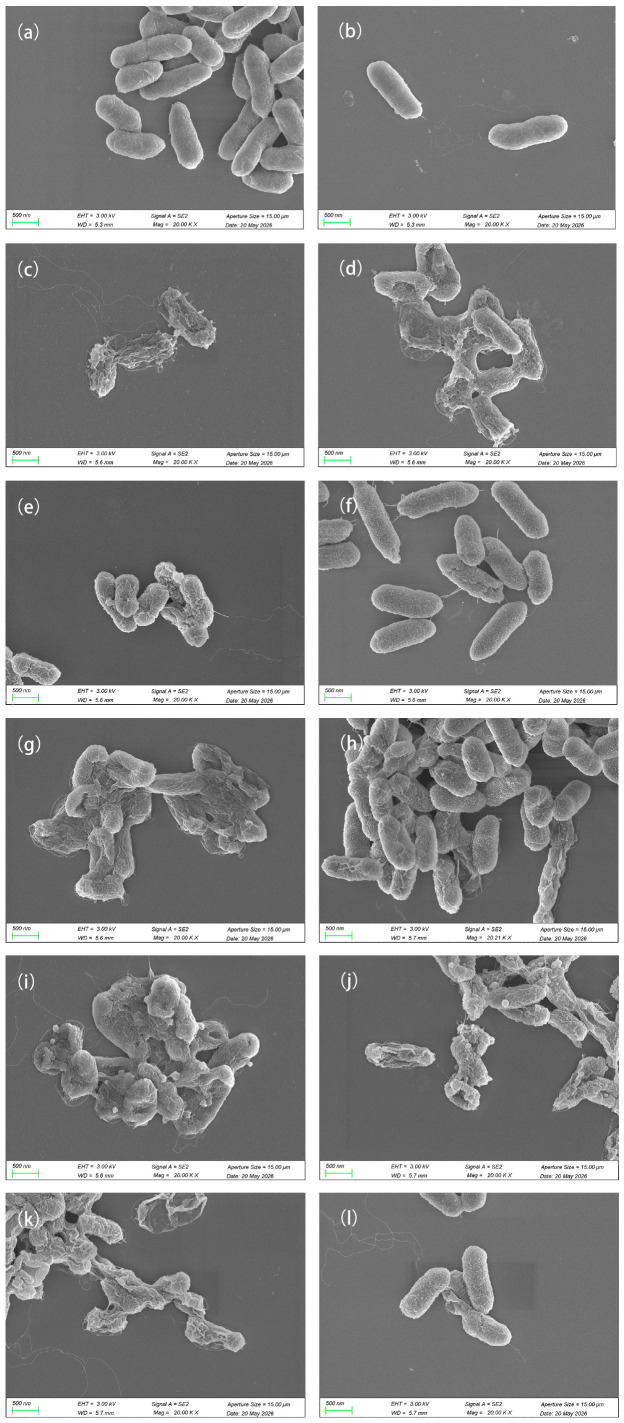
Effects of different concentrations of five plant essential oils on the cell morphology of *A. hydrophila*. (**a**,**b**): untreated control; (**c**,**d**): oregano essential oil treatment at 2 MIC and MIC; (**e**,**f**): cinnamon essential oil treatment at 2 MIC and MIC; (**g**,**h**): clove essential oil treatment at 2 MIC and MIC; (**i**,**j**): perilla essential oil treatment at 2 MIC and MIC; (**k**,**l**): rosemary essential oil treatment at 2 MIC and MIC.

Scanning electron microscopy (SEM) analysis, as illustrated in Figure 5, demonstrated that untreated *A. hydrophila* cells (panels a and b) preserved their typical rod-shaped morphology, exhibiting smooth surfaces and intact cellular structures. Following treatment with five distinct plant essential oils at a concentration corresponding to 1× the minimum inhibitory concentration (MIC) (panels d, f, h, j, and l representing oregano, cinnamon, clove, perilla, and rosemary essential oils, respectively), the bacterial cells displayed various surface alterations, including roughening, shrinkage, depressions, and morphological deformities. Elevating the concentration to 2× MIC (panels c, e, g, i, and k) induced more severe cellular damage, evidenced by cell collapse, rupture, compromised structural integrity, and the presence of cellular debris.

These observations suggest that all five plant essential oils disrupt the cellular morphology and structural integrity of *A. hydrophila*, with the severity of damage correlating positively with the concentration of the treatment. This implies that the antibacterial mechanisms of these essential oils may involve impairment of the bacterial cell wall and membrane structures. These findings align with previous studies by Zhong et al., Liang et al., and Sousa et al., which demonstrated that plant essential oils exert antibacterial effects against *A. hydrophila* by compromising membrane integrity, enhancing membrane permeability, and inducing leakage of intracellular components [35,36,37].

### 3.6. Application Prospects and Significance in Skipjack Tuna Preservation

In this study, a high histamine-producing strain of *Aeromonas hydrophila* isolated from skipjack tuna was employed as the target microorganism to assess the inhibitory effects of various plant essential oils on bacterial proliferation and histamine accumulation. Prior investigations have demonstrated that plant essential oils, such as oregano oil, can effectively suppress histamine-producing bacteria in tuna, underscoring their potential to mitigate histamine-related hazards in aquatic food products [38]. The present results offer further empirical support for the targeted control of specific histamine-producing bacteria through the application of plant essential oils and provide a basis for optimizing essential oil concentrations and delivery methods in seafood preservation [39,40].

It is critical to acknowledge that the practical efficacy of essential oils in actual food matrices may differ from in vitro observations. Variables including the composition of the food matrix, lipid and protein content, microstructural attributes, and processing conditions can substantially influence the diffusion, distribution, stability, and antimicrobial activity of essential oil constituents [41,42]. Moreover, the volatility, pronounced aroma, and sensory characteristics of essential oils may constrain their permissible usage levels in aquatic products [43,44].

From a practical standpoint, histamine formation in fish products can occur not only on the surface but also within muscle tissues, particularly in hypoxic or anaerobic microenvironments conducive to histidine decarboxylation [45,46]. Consequently, direct surface application of essential oils may not entirely prevent histamine accumulation if histamine-producing bacteria reside within the muscle or if the active compounds fail to sufficiently penetrate and distribute throughout the tissue [47]. Therefore, the preservative effectiveness of essential oils in skipjack tuna products is likely contingent upon their penetration capacity, interactions with muscle proteins and lipids, stability during storage, and sensory acceptability.

Beyond direct incorporation or surface treatment, vapor-phase application of essential oils may constitute a promising complementary preservation approach [48,49]. Given the volatility of many essential oil components, active packaging systems, absorbent pads, sachets, or edible coatings capable of controlled release of essential oil vapors could inhibit microbial growth while minimizing direct contact with fish muscle, thereby potentially reducing adverse sensory impacts [50]. Nonetheless, vapor-phase treatments may exhibit limited efficacy against histamine-producing bacteria located deep within muscle tissues. Accordingly, future research should undertake comparative evaluations of direct-contact and vapor-phase essential oil delivery systems within authentic skipjack tuna matrices under both refrigerated and temperature-abuse conditions, concurrently monitoring bacterial proliferation, histamine accumulation, histamine formation kinetics, sensory quality, and consumer acceptance.

## 4. Conclusions

In the present investigation, a bacterial strain exhibiting high histamine production was isolated from the dorsal muscle of skipjack tuna and identified as *A. hydrophila* through 16S rRNA gene sequencing analysis. This strain demonstrated substantial histamine synthesis in a histidine-enriched medium, suggesting its potential role in histamine accumulation under conducive environmental conditions.

The antibacterial efficacy of six plant-derived essential oils against this strain varied significantly. Oregano and cinnamon essential oils exhibited the most potent inhibitory effects, followed by clove essential oil, whereas perilla and rosemary essential oils showed comparatively moderate inhibition. Patchouli essential oil did not display notable antibacterial activity. Histamine accumulation was diminished in a concentration-dependent manner across the tested oils. At the minimum bactericidal concentration (MBC), all active essential oils completely suppressed histamine production. At half the MBC (MBC/2), oregano, cinnamon, and clove essential oils fully inhibited histamine synthesis, while perilla and rosemary essential oils significantly reduced it. Scanning electron microscopy (SEM) analyses revealed that treatment with essential oils induced damage to the bacterial cell surface and disrupted cellular morphology, indicating that structural compromise may underlie their antibacterial mechanisms.

Collectively, oregano and cinnamon essential oils emerged as the most promising natural antibacterial agents for mitigating high histamine-producing *A. hydrophila* isolated from skipjack tuna. Nonetheless, these results were primarily derived from in vitro culture systems. Therefore, further studies are warranted to validate their efficacy under actual skipjack tuna storage conditions, including assessments of microbial growth inhibition, histamine accumulation, sensory quality preservation, and safety considerations, prior to their practical application in aquatic product preservation.

## Figures and Tables

**Table 1:** Gradient Elution Program for HPLC Determination of Dansylated Histamine.

Time (min)	Mobile Phase A (%)	Mobile Phase B (%)
0.00	58	42
22.00	78	22
25.00	90	10
32.00	90	10
32.01	58	42
37.00	58	42

**Table 2:** Stock and final concentrations of plant essential oils prepared for the microdilution assay.

Essential Oil Stock Solution Concentration (mg/mL)	Volume Added (μL)	Medium Volume (μL)	Bacterial Suspension Volume (μL)	Total Volume of the Reaction System (μL)	Final Concentration (mg/mL)
20.0	100	850	50	1000	2.0
10.0	100	850	50	1000	1.0
8.0	100	850	50	1000	0.8
6.0	100	850	50	1000	0.6
4.0	100	850	50	1000	0.4
2.0	100	850	50	1000	0.2
1.0	100	850	50	1000	0.1

**Table 3:** Histamine production by bacterial strains isolated from skipjack tuna.

Isolated Strain	Histamine (mM)
AH-1-TU	0.1 ± 0.1
AH-2-TU	1.2 ± 0.2
AH-3-TU	n.d.
AH-4-TU	n.d.
AH-5-TU	0.3 ± 0.1
AH-6-TU	n.d.
AH-7-TU	n.d.

Note: Histamine production was quantified following incubation of the strains in nutrient broth (NB) supplemented with 1% (*w*/*v*) histidine at 30 °C for five days. Data are presented as mean ± standard deviation (SD) from three independent biological replicates (*n* = 3). n.d., not detected.

**Table 4:** Molecular identification of strain AH-2-TU based on 16S rRNA gene sequencing and BLAST analysis.

Item	Result
Strain	AH-2-TU
16S rRNA sequence length	1537 bp
GenBank accession no.	NR_074841.1
Closest match	*Aeromonas hydrophila* ATCC 7966
Query coverage	99%
Percentage identity	99.86%
E-value	0.0
Phylogenetic placement	clustered with *A. hydrophila* reference strain

**Table 5:** Inhibition zone diameters of six plant essential oils against *A. hydrophila*.

Plant Essential Oil	Halo Inhibition on *A. hydrophila* (Strain)
Oregano essential oil	27 ± 0.58 ^a^
Perilla essential oil	9 ± 0.29 ^d^
Cinnamon essential oil	25 ± 1.15 ^b^
Rosemary essential oil	9 ± 0.29 ^d^
Clove essential oil	14 ± 0.58 ^c^
Patchouli essential oil	-
Negative control (Tween 80, 1%, *v*/*v*)	-

Note: -: no inhibition halo. Different superscript letters indicate significant differences (*p* < 0.05).

**Table 6:** Minimal inhibitory concentration (MIC), minimal bactericidal concentration (MBC), and MBC/MIC ratios of the most effective antimicrobial compounds against *A. hydrophila* (strain AH-2-TU).

Plant Essential Oil	MIC (mg/mL)	MBC (mg/mL)	MBC/MIC
Oregano essential oil	0.2	0.4	2.0
Perilla essential oil	1.0	2.0	2.0
Cinnamon essential oil	0.1	0.4	4.0
Rosemary essential oil	2.0	4.0	2.0
Clove essential oil	0.4	1.0	2.5

## Data Availability

The original contributions presented in this study are included in the article. Further inquiries can be directed to the corresponding author.
